# Seroprevalence of SARS-CoV-2 IgG antibodies in a healthcare setting during the first pandemic wave in Senegal

**DOI:** 10.1016/j.ijregi.2021.12.008

**Published:** 2021-12-24

**Authors:** Ambroise D. Ahouidi, Mark Anderson, Cyrille K. Diédhiou, Aminata Dia, Moustapha Mbow, Yacine Dia, Aminata Mboup, Astou Gueye Gaye, Noel M. Manga, Gavin Cloherty, Souleymane Mboup

**Affiliations:** a Institut de Recherche en Sante, de Surveillance Epidemiologique Et de Formations, Arrodissement 4 Rue 2 D1, Pole Urbain de Diamiadio, BP 7325, Dakar, Senegal; b Abbott Laboratories, Infectious Disease Research, Abbott Park, IL, USA; c Unit of Infectious and Tropical Diseases, Assane Seck University, Hospital de la Paix, Ziguinchor, Senegal

**Keywords:** Serosurvey, SARS-Cov-2, healthcare, IgG, Senegal

## Abstract

This study investigated the seroprevalence of severe acute respiratory syndrome coronavirus-2 (SARS-CoV-2) immunoglobulin G (IgG) during the first pandemic wave in Senegal. The seroprevalence rate of SARS-CoV-2 IgG was assessed in 10 cities in Senegal by testing plasma from volunteers attending healthcare clinics for reasons unrelated to coronavirus disease 2019 (*n*=3231) between June and October 2020. The overall positivity rate was 20.4% and large geographical differences in seropositivity (6–41.9%) were observed, suggesting that the true number of infections was substantially higher than the official estimate of 8.5%.

## Highlights

• The seroprevalence of severe acute respiratory syndrome coronavirus-2 immunoglobulin G in 10 cities in Senegal was investigated by testing plasma.
• Differences in seropositivity were observed between cities.
• The number of cases of coronavirus disease 2019 in Senegal was higher than the number of cases reported during the first wave of the pandemic.

## Introduction

The first wave of severe acute respiratory syndrome coronavirus-2 (SARS-CoV-2), the causative agent of coronavirus disease 2019 (COVID-19), reached Africa in February 2020. In low-income countries such as Senegal, critical public health interventions such as social distancing and lockdowns are difficult due to the enormous impact on livelihoods. Senegal recorded the first case of COVID-19 on 3 March 2020. At the time of this study, the Senegalese Ministry of Health estimated the national prevalence of SARS-CoV-2 to be 8.5% based on polymerase chain reaction testing of symptomatic patients, close contacts and international travellers (Ministere de la Sante et de l'action Sociale, 2021).

This article reports SARS-CoV-2 seroprevalence data from 3231 individuals across 10 cities in Senegal collected between June and October 2020. An adjusted prevalence rate of SARS-CoV-2 immunoglobulin G (IgG) of 20.4% was identified. The striking difference between the rate in this study and the official estimate may be explained by the lack of testing capacity, asymptomatic or mild cases, and willingness to be tested due to social stigma associated with COVID-19. Additionally, the seroprevalence of SARS-CoV-2 IgG was found to be unequally distributed across the country, with the highest rates found in Dakar (20.6%) and Ziguinchor (41.7%), and the lowest rates found in Louga (5.7%) and Tambacounda (8.7%).

Plasma was collected from 3231 volunteers who attended health clinics in 10 cities in Senegal between June and October 2020 for reasons other than symptoms of COVID-19 (e.g. fever, cough, stiffness, loss of smell or taste), and included individuals accompanying patients to clinics (Table 1). The study setting was chosen due to the availability of participants during the early lockdown in the first pandemic wave. Demographic data, including age, sex and area of residence, were collected for each participant. Participant age ranged from 1 to 91 years [median 37 years, 25th and 75th percentiles of 25 and 55, respectively; mean 41.1 (standard deviation 18.2) years].

**Table 1:** Seroprevalence of severe acute respiratory syndrome coronavirus-2 by location, sex and age.

Location/group	Prevalence *n* (%)	Total sampled	Adjusted prevalence	2020 estimated population	Expected infections^a^
Dakar	178 (20.9)	852	20.6	3,835,011	790,252
Thiès	35 (18.3)	191	18.0	2,162,833	389,236
Ziguinchor	156 (41.9)	372	41.7	683,955	285,101
Saint-Louis	78 (19.7)	396	19.4	1,091,735	211,816
Kaolack	39 (14.5)	269	14.2	1,191,577	168,995
Kolda	79 (19.9)	397	19.6	822,003	161,133
Touba	70 (17)	395	16.7	529,176	88,329
Tambacounda	12 (9)	133	8.7	872,155	75,546
Louga	6 (6)	100	5.7	1,061,612	59,991
Bounkiling	15 (11.9)	126	11.6	145,568	16,846
Total	668 (20.7)	3231	20.4	16,705,608	3,408,863
Age (years)					
0–14	2 (18.2)	11	17.9	6,955,469	1,244,766
15–29	235 (18.9)	1241	18.6	4,550,798	846,395
30–44	164 (17.2)	955	16.9	2,791,905	471,623
45–59	129 (24.3)	530	24.0	1,480,565	355,615
60–74	108 (27.6)	392	27.3	717,929	196,218
>75	30 (29.4)	102	29.1	208,942	60,881

a Expected infections = (seropositivity*population 2020)/100.

Samples were tested on an Abbott ARCHITECT *i*1000SR with CE-marked SARS-CoV-2 IgG according to the instructions on the package insert. Briefly, the SARS-CoV-2 IgG assay is an automated chemiluminescent microparticle immunoassay that qualitatively detects IgG antibodies directed against the SARS-CoV-2 nucleocapsid protein, and index values ≥1.4 S/C indicate a positive result. Specificity was 99.63% and sensitivity of known infections was calculated to be 100% 2 weeks after infection based on the package insert. Prevalence data were adjusted for sensitivity and specificity. Differences in the proportion of IgG-positive cases between cities were assessed using Pearson's Chi-squared test. Fisher's exact test was used to determine if differences in the frequency of IgG between age groups were statistically significant. *P*<0.05 was considered to indicate significance. Estimated infections by location and age were calculated by multiplying the adjusted prevalence rate by the estimated population in 2020 (Diop et al., 2021) and divided by 100.

**Figure 1:**
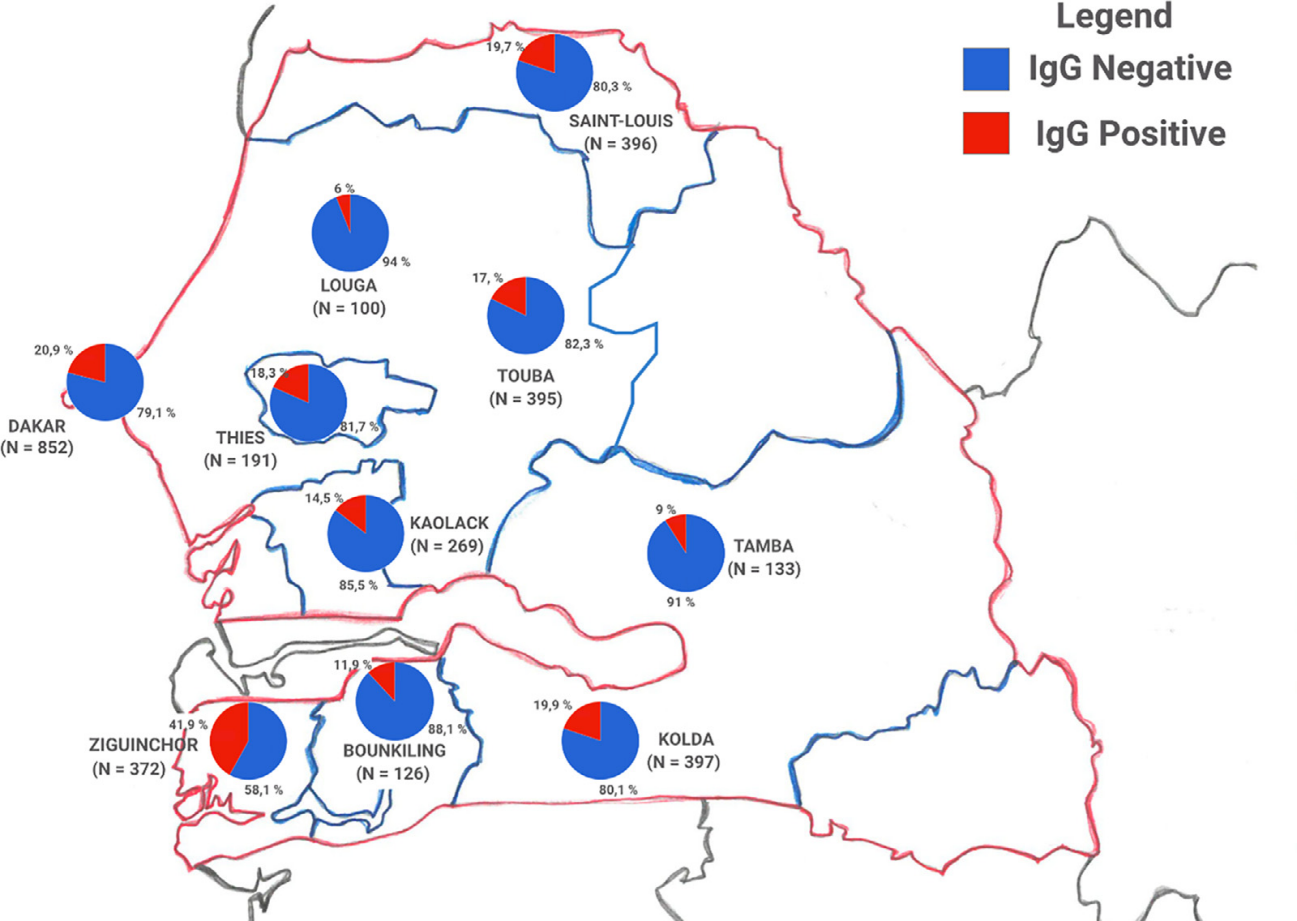
Sample collection sites and seroprevalence of severe acute respiratory syndrome coronavirus-2 immunoglobulin G (IgG) antibodies across Senegal.

The adjusted prevalence rate of SARS-CoV-2 IgG among all tested participants was 20.4%, and this ranged widely (5.7–41.7%; median 18%) between locations (Table 1 and Figure 1). Ziguinchor had the highest seroprevalence rate (41.7%) among all sites. Dakar had a positive sample rate of 20.6% and was similar to Thies (18.0%), Saint-Louis (19.4%), Touba (16.7%) and Kolda (19.6%). Areas with lower rates of positivity were Louga (5.7%), Kaolack (14.2%), Tambacounda (8.7%) and Bounkiling (11.6%). Differences in SARS-CoV-2 IgG prevalence between cities were significant (*P*<0.001).

Stratifying SARS-CoV-2 seropositivity by age group (1–14, 15–29, 30–44, 45–59, 60–74 and >75 years) showed no age-dependent differences in seroprevalence in individuals aged <44 years (*P*=0.534). However, steadily increasing positivity was identified in the older age groups, peaking at 29.1% in subjects aged >75 years, similar to observations in studies conducted elsewhere (Sasson et al., 2021). An age-dependent difference in seroprevalence was found when the test was applied to all age groups, possibly suggesting stronger immune responses in older patients or potentially faster seroreversion in younger patients (*P*<0.001).

This investigation provides insights into the first wave of the COVID-19 pandemic in Senegal, where very few positive cases (*n*=70,125) have been reported using PCR-based testing compared with the expected 3.4 million infections based on this seroprevalence study and the size of the population of Senegal in 2020. Similarly, the 20.4% positivity rate calculated in this study is much higher than the rate of 8.5% that was estimated by the Senegalese Ministry of Health at the time when this study was conducted. This is likely explained by extremely limited testing for COVID-19 even for symptomatic and suspected cases. Furthermore, SARS-CoV-2 seroprevalence varied significantly between geographical regions of Senegal (*P*<0.001), and was particularly high in the southern city of Ziguinchor. This region of Senegal is bordered by three countries (The Gambia, Guinea and Guinea Bissau), and substantial cross-border travel occurs in this area. Official reports on the percentages of the total populations that have been infected in The Gambia, Guinea and Guinea Bissau are 0.39%, 0.22% and 0.32%, respectively (Roca, 2020; World Health Organization, 2021). These low prevalence numbers likely do not represent the total number of infections in these countries, as demonstrated for Senegal in the present study, and similar serosurveys conducted in these countries may identify higher numbers of prior infections. A limitation of this study is that the population studied consisted of individuals who attended clinics for non-COVID-19-related symptoms, and may not be fully representative of the general population of Senegal. In addition, pre-pandemic samples were unavailable to validate the SARS-CoV-2 IgG assay in the general Senegalese population. These results suggest that the spread of COVID-19 in Senegal was far higher than indicated by the official estimate during the first wave of infections in mid–late 2020. Follow-up prevalence studies should be performed, particularly in the light of highly transmissible variants, to assess the level of herd immunity and develop a vaccine strategy in Senegal.

## Funding

This research was supported by Abbott Laboratories and in part through National Institutes of Health UAS grant U01 AI151698 for the United World Antivirus Research Network (UWARN).

## Ethical approval

The study was reviewed and approved by the Ethical Committee of the Ministry of Health of Senegal (000129/MSAS/CNERS). All participants gave informed written consent.

## Declaration of Competing Interest

None declared.
